# STAR Caregivers: Virtual Training and Followup: Implementation

**DOI:** 10.1093/geroni/igab046.530

**Published:** 2021-12-17

**Authors:** Robert Penfold, Maggie Ramirez, Susan McCurry, Linda Terry, James Ralston, Kelly Hansen

**Affiliations:** 1 Kaiser Permanente Washington Health Research Institute, Seattle, Washington, United States; 2 University of Washington School of Public Health, Seattle, Washington, United States; 3 University of Washington School of Nursing, Seattle, Washington, United States

## Abstract

STAR Caregivers is an evidence-based intervention designed to reduce caregiver burden for caregivers of people living with dementia. This study translated the paper-based, face-to-face intervention into a 6-session, self-directed online learning program supported by 6, 30-minute telephone calls with a clinically trained coach. Our approach is designed to overcome issues of access to training. Eligible caregiver-patient dyads at Kaiser Permanente Washington were identified automatically via electronic health records. Qualitative interviews were conducted with a sample of patients to elicit information about their needs and preferences for training. We developed a “learning management system” (analogous to compliance training modules) complete with slides, voice-over narration, and testing. The training sessions are mounted on the KP Learn site and accessible to anyone. Baseline and outcomes data on standardized instruments are collected remotely via REDCap. Recruitment for the study is ongoing and initial participant feedback on the program is very positive.

